# Classifying natural products from plants, fungi or bacteria using the COCONUT database and machine learning

**DOI:** 10.1186/s13321-021-00559-3

**Published:** 2021-10-18

**Authors:** Alice Capecchi, Jean-Louis Reymond

**Affiliations:** 1 Department of Chemistry, Biochemistry and Pharmaceutical Sciences, University of Bern, Freiestrasse 3, 3012 Bern, Switzerland

**Keywords:** Natural products, Cheminformatics, Chemical space, Visualization, Molecular fingerprints, Machine learning, Support vector machine

## Abstract

Natural products (NPs) represent one of the most important resources for discovering new drugs. Here we asked whether NP origin can be assigned from their molecular structure in a subset of 60,171 NPs in the recently reported Collection of Open Natural Products (COCONUT) database assigned to plants, fungi, or bacteria. Visualizing this subset in an interactive tree-map (TMAP) calculated using MAP4 (MinHashed atom pair fingerprint) clustered NPs according to their assigned origin (https://tm.gdb.tools/map4/coconut_tmap/), and a support vector machine (SVM) trained with MAP4 correctly assigned the origin for 94% of plant, 89% of fungal, and 89% of bacterial NPs in this subset. An online tool based on an SVM trained with the entire subset correctly assigned the origin of further NPs with similar performance (https://np-svm-map4.gdb.tools/). Origin information might be useful when searching for biosynthetic genes of NPs isolated from plants but produced by endophytic microorganisms.

**Supplementary Information:**

The online version contains supplementary material available at 10.1186/s13321-021-00559-3.

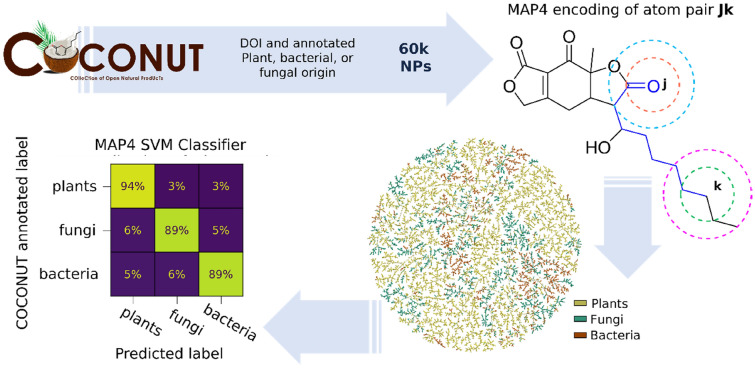

## Introduction

Due to the importance of natural products (NPs) in drug discovery [1, 2], there is a considerable interest in describing and understanding their structural diversity, particularly by exploiting NP databases [3] using in silico methods such as machine learning (ML) [4]. Computational approaches have been reported to distinguish between NPs and non-NPs [5–9], between terrestrial and marine NPs [10], and to classify NP structural types [11, 12] and visualize their chemical space [13].

In our own approach to this problem [14], we recently analyzed NPAtlas, an open-access database listing 25,523 NPs from bacterial or fungal origin [15], by computing the MAP4 fingerprint (MinHashed Atom-Pair fingerprint up to four bonds) [16] of each NP and creating a TMAP (tree-map) [17] of the resulting high-dimensional dataset. In this analysis, NPs from bacterial or fungal origin formed separated clusters. This separation effect was confirmed by showing that a support vector machine (SVM) trained with the MAP4 of NPAtlas was able to distinguish bacterial or fungal origin, including a recently reported NP isolated from the marine sponge *Phakellia fusca* assigned by our classifier to be of bacterial origin, in line with the fact that many NPs from this sponge originate from endosymbiotic actinobacteria [18, 19].

The possibility to assign the origin of NPs from their structure was intriguing because most NPs are secondary metabolites produced by biosynthetic gene clusters [20] which are sometimes transferred between different organisms [21]. Such horizontal gene transfer may reflect adaptative relationships between host organisms such as plants and sponges and endosymbiotic bacteria or fungi [22]. Among the many endophytic NPs [23, 24], striking examples include the cancer drug paclitaxel, a plant NP also produced by endophytic fungi of the yew tree [25, 26], and maytansine, used in antibody-drug conjugates for cancer therapy and produced by endophytic bacteria in plants [27]. Due to the very widespread occurrence of endophytic bacteria and fungi in plants, we asked whether our MAP4 analysis might be able to distinguish plant NPs from bacterial and fungal NPs. To test this hypothesis, we considered the recently reported COCONUT database, an open-access database currently offering the most extensive coverage and including plant NPs [28].

## Results and discussion

### Chemical space analysis of plant and microbial NPs from the COCONUT database

COCONUT collects over 400 thousand NPs from 52 different databases, 135 thousand of which are annotated with a taxonomical origin. For our analysis, we considered the 68 thousand entries annotated with a source organism that were also associated with a publication. We focused on those annotated as originating from plants (50%), fungi (23%), or bacteria (16%), leaving out a smaller subset of NPs originating from animals (2%), homo sapiens (2.5%), of marine origin (1.5%), or lacking one of the previous taxonomical annotations (5%). The selected subset of 60,171 NPs contained 33,772 plant NPs, 15,648 fungal NPs and 10,751 bacterial NPs.

The subset spanned from molecular weight MW = 81 Da for 1,2-dihydropyridine, a plant NP [29], to MW = 2901 Da for lacticin 481, a bacterial peptide [30]. Plant NPs dominated the intermediate molecular weight range (200 < MW < 800), while fungal NPs were most abundant in the low molecular weight range (MW ≤ 200) and bacterial NPs in the high MW range (MW ≥ 800). The three series had rather similar distributions of the fraction of sp^3^ carbon atoms (Fsp3), which measures the degree of saturation. However, the estimated octanol:water partition coefficient AlogP indicated that highly polar NPs were almost absent from fungal NPs. Furthermore, plant NPs had overall higher percentages of glycosides, while peptides were almost absent from plant NPs and most abundant in bacterial NPs (Table 1).

**Table 1:** Property distribution and origin of the 60,171 COCONUT entries with a DOI and annotated as plants, fungal, or bacterial

	Plants NPs^a^	Fungal NPs^a^	Bacterial NPs^a^
MW ≤ 200^b^	7072 (21%)	4919 (31%)	2237 (21%)
200 ≤ MW < 800^b^	24,078 (71%)	10,111 (65%)	6066 (56%)
MW ≥ 800^b^	2622 (8%)	618 (4%)	2448 (23%)
Fsp3 ≤ 0.2^c^	4213 (13%)	1580 (10%)	1073 (10%)
0.2 ≤ Fsp3 < 0.8^c^	22,032 (65%)	11,334 (72%)	7986 (74%)
Fsp3 ≥ 0.8^c^	7527 (22%)	2734 (18%)	1692 (16%)
AlogP ≤ − 2^d^	4855 (14%)	373 (2%)	1446 (13%)
− 2 ≤ AlogP < 8^d^	28,315 (84%)	15,000 (96%)	8906 (83%)
AlogP ≥ 8^d^	602 (2%)	275 (2%)	399 (4%)
Glycosides^e^	8260 (24%)	797 (5%)	1793 (17%)
Peptides^f^	194 (<1%)	676 (4%)	2053 (19%)

^a^COCONUT entries with a DOI and the specified taxonomical origin annotated; percentages refer to the total number of the selected entries within the specified class: 33,772 plants NPs, 15,648 fungal NPs, and 10,751 bacterial NPs

^b^Molecular weight (MW) calculated with RDKit

^c^Fraction of sp3 (Fsp3) calculated with RDKit

^d^Octanol: water partition coefficient calculated with RDKit following the Crippen method (AlogP)

^e^Containing a cyclic *N*- or *O*-acetal substructure defined through SMARTS language

^f^Containing a dipeptide substructure defined through SMARTS language

To get a closer insight into structural features, we calculated the MAP4 fingerprint for each of the 60,171 selected NPs. MAP4 encoding combines the characteristics of substructure fingerprints, which are well suitable for small molecules, and of atom pair fingerprints, which are instead preferable for larger structures, and it has been proven suitable for both [16]. It consists of listing all pairs of circular substructures of radius 1 and 2 as SMILES, separated by their topological distance in bonds, and MinHashing the resulting set of SMILES pairs to a defined dimensionality (1024 in the present analysis). We then represented the MAP4 annotated NP dataset using the dimensionality reduction method TMAP. This method is suitable for very large high-dimensional datasets and performs better than t-SNE or UMAP in preserving local and global relationships in the data [17]. To create a TMAP, the algorithm computes an approximate nearest neighbor graph by locality sensitive hashing (LSH), cuts edges to obtain the minimum spanning tree of this graph, and creates an optimized 2D representation of the minimum spanning tree, in which each node represent a molecule connected to its approximate nearest neighbors. This tree is then displayed with interactive the visualization tool Faerun [31]. Faerun shows each node as a sphere that can be color-coded according to various properties and uses Smilesdrawer [32] to depict molecular structures. The TMAP of our NP subset is available interactively at https://tm.gdb.tools/map4/coconut_tmap/.

**Fig. 1:**
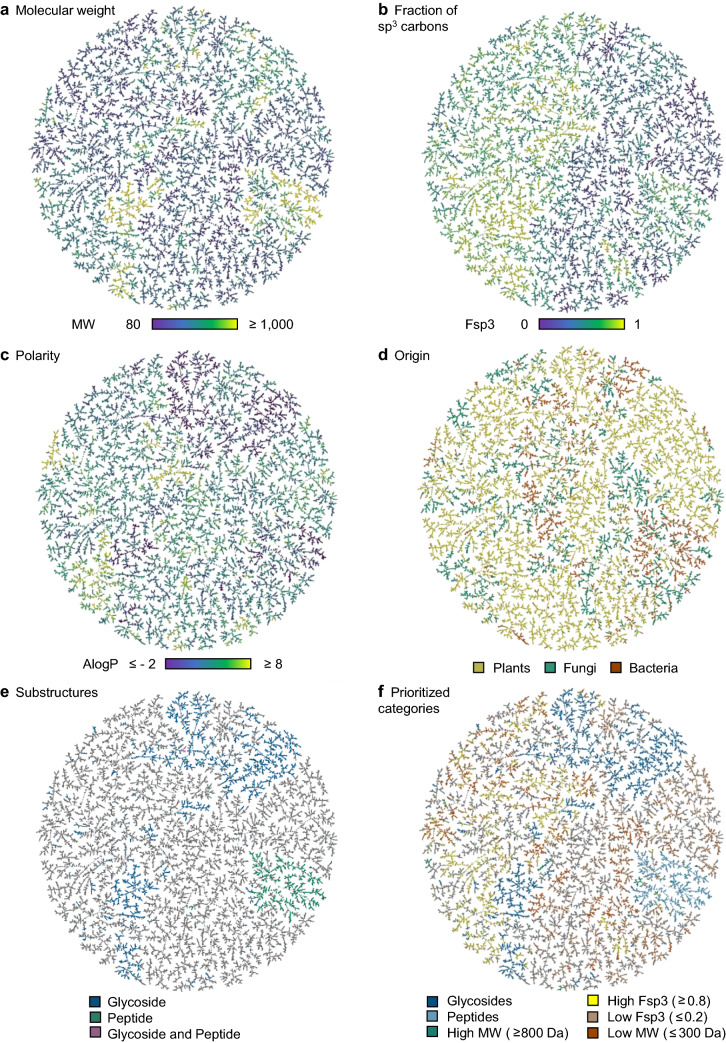
MAP4 TMAP of the 60 thousand selected COCONUT entries. The maps are colored according to **a** molecular weight MW in Da, **b** fraction of sp3 carbon atoms Fsp3, **c** calculated octanol:water partition coefficient AlogP, **d** COCONUT annotated origin, **e** presence of a glycoside (blue) or peptide (green) substructure, or both (magenta), **f** prioritized categories: glycosides (entries containing a glycoside substructure, blue) > peptides (entries containing a peptide substructure, cyan) > high MW (green) > high Fsp3 (yellow) > low Fsp3 (orange) > low MW (red). Entries not belonging to any category are reported in gray. All maps are accessible in an interactive format at https://tm.gdb.tools/map4/coconut_tmap/

The TMAP of our NP subset color-coded by MW showed that most high MW compounds appeared in two groups, the first one (at right on the TMAP), contained peptides and related macrocycles, and the second one (at middle/lower left on the TMAP) corresponded to glycosylated triterpenoids (Fig. 1a). Color-coding by Fsp3 showed that the TMAP separated high Fsp3 molecules (left half of the TMAP), comprising many terpenes, steroids, and glycosides, from low Fsp3 molecules (right half of the TMAP) featuring many polyphenols and related polyaromatic molecules (Fig. 1b). Furthermore, the color-code by the calculated octanol:water partition coefficient AlogP, estimating polarity, showed several islands of highly polar NPs (low AlogP, magenta) corresponding mostly to nucleosides and glycosylated polyphenols (upper part of the TMAP), glycosylated triterpenoids (lower left on the TMAP) and peptides (middle right on the TMAP), as well as a few groups of apolar NPs (high AlogP, red), corresponding primarily to lipids, terpenes, and steroids (Fig. 1c)

Color-coding by the annotated origin showed that NPs from plants, fungi, or bacteria formed many well-defined clusters spread across the entire TMAP (Fig. 1d). On the one hand, this separation illustrated how NP origin corresponded to differences in molecular structure that were well perceived by the MAP4 fingerprint used to generate the map. On the other hand, the taxonomical origin color code also showed that each subset contained diverse structural types. While there was no correlation of origin with properties such as MW, Fsp3, or AlogP, most glycosides were associated with plants, and most peptides were of bacterial or fungal origin, in line with Table 1 (Fig. 1e). These relationships were also well visible by color-coding the TMAP by six selected prioritized categories summarizing important characteristics of natural products (Fig. 1f)

### Statistical modeling of NP origin using support vector machines (SVM)

The clear separation of NPs from plants, fungi, or bacteria in the TMAP above clearly showed that our MAP4 fingerprint distinguished between NPs of plant, bacterial or fungal origin. To further investigate this separation, we trained an SVM classifier using the MAP4 similarity matrix of half of the COCONUT subset and used the other half to evaluate it. Indeed, the obtained MAP4 SVM correctly predicted the origin of 94% of plant NPs, 89% of fungal NPs, and 89% of the bacterial NPs (MAP4 SVM), resulting in a balanced accuracy of 0.897, an MCC (Matthews correlation coefficient) of 0.890, and an F1 score of 0.920 (see Methods for a detailed explanation of the used metrics).

To better identify the role of the MAP4 molecular encoding in the reported successful prediction, we compared the performances of a MAP4 SVM with the performances of an SVM trained using ECFP4 (Extended Connectivity Fingerprint ECFP of radius 2, ECFP4 SVM) and the RDKit atom pair fingerprint (AP SVM). We chose ECFP4 and the RDKit AP as widely used and available examples of substructures fingerprints and atom pair fingerprints. As a baseline model, we also included an SVM trained with a set of 11 calculated physico-chemical properties, namely MW, Fsp3, HBD (hydrogen bond donor) count, HBA (hydrogen bond acceptor) count, AlogP, the number of carbons, oxygens, and nitrogens, the total number of atoms, number of bonds, and TPSA (topological polar surface area) (properties SVM). The selected 60 thousand COCONUT entries were divided into five subsets, and each model was trained and evaluated five times using the five different 80-20 training test splits combinations of one subset as test set and the other four as training set. Then the mean balanced accuracy, MCC, and F1 score of the five evaluations were calculated.

**Fig. 2:**
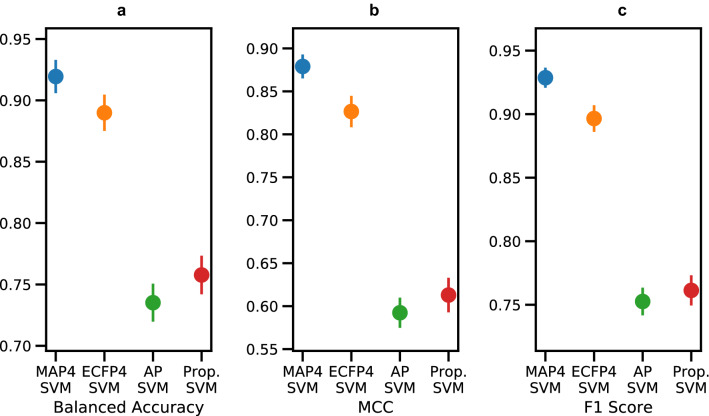
Fivefold cross-validation mean values and 3σ confidence intervals of the **a** balanced accuracy, **b** MCC, and **c** F1 score for the four SVM classifiers. In all panels, the MAP4 SVM is reported in blue, the ECFP4 SVM in orange, the RDKit AP (AP) SVM in green, and the properties (Prop.) SVM in red

The results of this evaluation are presented in Table 2; Fig. 2. Remarkably, all four SVM performed reasonably well. The good performance of the property based SVM reflected the fact that relatively large NP families with characteristic properties are essentially all from the same origin. For example, almost all large peptides or cyclic peptides are assigned to bacteria, while most glycosylated triterpenoids and polyphenols are assigned to plants. Nevertheless, there was a significant performance increase with the ECFP4 SVM and MAP4 SVM, which performed best, showing that correct origin assignment works better if specific substructures are considered. Among the four SVM evaluated, our MAP4 SVM performed best with significantly higher values compared to the ECFP4 SVM, probably because the MAP4 fingerprint encodes a more precise representation of the molecular structures than ECFP4. Indeed, MAP4 considers pairs of local substructures and the topological distance between them, while ECFP4 only encodes the presence of local substructures.

**Table 2:** SVM evaluation with balanced accuracy, MCC, and F1 score

	Balanced acc.	MCC	F1
MAP4 SVM ^a,b^	0.919 ± 0.005	0.879 ± 0.005	0.929 ± 0.003
ECFP4 SVM ^a,b^	0.890 ± 0.005	0.827 ± 0.006	0.897 ± 0.003
RDKit AP SVM ^a,b^	0.735 ± 0.005	0.592 ± 0.006	0.752 ± 0.004
Properties SVM ^a,c^	0.758 ± 0.005	0.613 ± 0.007	0.761 ± 0.004

^a^Mean value and standard deviation (σ) of the five different test/training sets split of the fivefold cross-validation

^b^1024 dimensions

^c^11 properties: MW, Fsp3, HBD) and HBA, calculated logP with the Crippen method (AlogP), number of carbons, oxygen, and nitrogen, the total number of atoms, number of bonds, and topological polar surface area (TPSA)

### Using the MAP4 SVM to assign the origin of NPs

The SVM evaluation above showed that the MAP4 analysis of NP molecular structure identified features distinguishing between NPs assigned to plants, fungi, and bacteria. Assuming that most of the assigned origins were correct among the 60,171 NPs used for training, one may use an SVM to tentatively assign the origin of further NPs as originating from plants, fungi, or bacteria. To best exploit the information in the COCONUT database, we trained a MAP4 SVM using the entire set of 60 thousand COCONUT NPs assigned to plants, fungi, or bacteria. We used the resulting classifier to build an online tool that takes any molecular structure as input (drawn or pasted as SMILES) and returns the assigned origin and the corresponding percentages from the SVM classifier. This tool is freely accessible online at https://np-svm-map4.gdb.tools/.

**Fig. 3:**
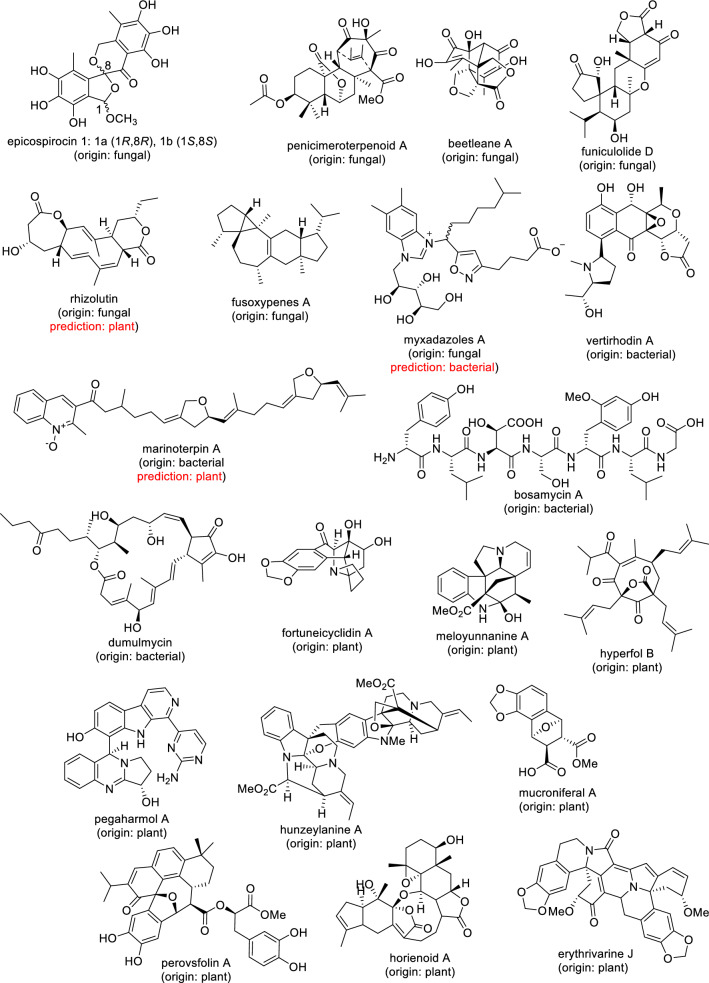
Chemical structure of 20 recently published microbial and plants NPs which are not present in COCONUT. The MAP4 SVM prediction is identical with the origin unless marked otherwise

The online tool performed quite well in assigning the origin of newly published NPs which were not present in COCONUT. Among 20 recently reported NPs from plants, fungi, or bacteria, 17 were correctly assigned to their origin, while only three were misassigned (Table 3; Fig. 3). In details, the fungal epicospirocin 1 [33], penicimeroterpenoid A [34], beetleane A [35], funiculolide D [36], and fusoxypenes A [37], the bacterial vertirhodin A [38], bosamycin A [39], and dumulmycin [40], and the plant fortuneicyclidin A [41], meloyunnanine A [42], hyperfol B [43], pegaharmol A [44], hunzeylanine A [45], mucroniferal A [46], perovsfolin A [47], horienoid A [48], and erythrivarine J [49] were correctly classified. On the other hand, the fungal rhizolutin [50] and myxadazoles A [51] and the bacterial marinoterpin A [52] were misclassified. Note that in these cases, the percentage values to the assigned class were lower than for the correct predictions.

**Table 3:** MAP4 SVM origin prediction for 20 recently published microbial and plants NPs that are not present in COCONUT

Natural product	Origin	MAP SVM prediction^a^
Epicospirocin 1	Fungal	Fungal (97%)
Penicimeroterpenoid A	Fungal	Fungal (82%)
Beetleane A	Fungal	Fungal (97%)
Funiculolide D	Fungal	Fungal (85%)
Rhizolutin	Fungal	Plant (55%, fungal: 29%)
Fusoxypenes A	Fungal	Fungal (69%)
Myxadazoles A	Fungal	Bacterial (74%, fungal: 16%)
Vertirhodin A	Bacterial	Bacterial (88%)
Marinoterpin A	Bacterial	Plant (44%, bacterial: 37%)
Bosamycin A	Bacterial	Bacterial (94%)
Dumulmycin	Bacterial	Bacterial (80%)
Fortuneicyclidin A	Plant	Plant (98%)
Meloyunnanine A	Plant	Plant (99%)
Hyperfol B	Plant	Plant (93%)
Pgaharmol A	Plant	Plant (77%)
Hunzeylanine A	Plant	Plant (95%)
Mucroniferal A	Plant	Plant (73%)
Perovsfolin A	Plant	Plant (92%)
Horienoid A	Plant	Plant (95%)
Erythrivarine J	Plant	Plant (91%)

^a^Predicted using the MAP4 SVM available online at https://np-svm-map4.gdb.tools/

As an additional test of our online tool, we investigated the predicted origin of the 3364 NPs (Additional file 1) in COCONUT reported with an origin and a publication for which the organism name was reported (e.g. *Brachystemma calycinum*) but not the corresponding taxonomical annotation as plant, fungi, bacteria. Checking individual predictions showed that the predicted origin was in many cases correct, in line with our performance evaluation. For example, the 49 NPs with *Euphorbia* as a source, many of which were peracetylated polycyclic terpene alcohols, as well as the 45 NPs with *Radula* as a source, which were polyphenols and terpenes, were all correctly assigned to a plant origin.

In several cases, the SVM prediction conflicted with the taxonomy of the reported source organism. For example, the indole alkaloids cephalinones A-D and cephalandoles A-C isolated from the orchid *Cephalanceropsis gracilis* [53] and whose structures were partly revised by total synthesis [54], were all assigned to bacteria by our SVM. In fact, These NPs might stem from an endophytic bacterium considering that endophytic microorganisms produce several related indole alkaloids [55]. Our SVM also reassigned the cancer drug maytansin from an annotated plant origin in the training set to a predicted bacterial origin, in line with its endophytic origin [27]. On the other hand, our classifier also assigned a bacterial origin to two cyclic peptides (CNP0085258 and CNP0085259) [56] and a cyclotide (CNP0085363) [57] isolated from plants. Although these plants indeed contain endophytic bacteria, the plant origin of such peptides is well established [58, 59], and the SVM assignment to bacteria reflects the fact that the majority of cyclic peptides and cyclotides in the COCONUT set used for training the SVM were assigned to bacteria, compared to only a handful of cyclotides of plant origin.

While the classifier may point to the possible endophytic origin of NPs isolated from plants, its use on NPs from other sources is problematic. For instance, among the 1,035 marine NPs from COCONUT with an annotated origin, 639 were assigned to plants by our SVM. This prediction must be mostly wrong considering that most marine organisms such as algae, corals, and sponges are not plants. For example, the 44 NPs from the soft coral *Sinularia*, or the macrocyclic terpene lactone lobophytolide A (CNP0275045) stemming from the soft corral *lobophytum cristagalli *[60, 61], were all incorrectly assigned to plants. However, the remaining 231 fungal and 165 bacterial predictions might be partly correct considering that many marine organisms carry endosymbionts. For example, our classifier assigned a bacterial origin for echinosulfonic acid B (CNP0318329), a brominated bis-indole NP isolated from the marine sponge *Echinodictyum gorgonoides *[62]. In this case, other authors have reported the isolation of a bacterial strain from the same sponge as a probable source of its biological activities [63].

## Conclusions

In summary, we visualized the chemical space covered by a subset of 60 thousand NPs from the COCONUT database with an assigned origin and publication using a TMAP calculated on the basis of MAP4 as molecular fingerprint, which is available at https://tm.gdb.tools/map4/coconut_tmap/. Analyzing this TMAP revealed that NPs from plant, fungal or bacterial origin form well separated groups. We then trained an SVM classifier with the MAP4 fingerprint to assign the origin of NPs and found that it performed excellently and significantly better than classifiers trained with ECFP4, RDkit AP, or physico-chemical descriptors.

To help assign NP origin, we then trained a MAP4 SVM classifier using the entire set of 60 thousand NPs. This tool is available online at https://np-svm-map4.gdb.tools/ and returns an origin prediction for any molecular structure drawn or pasted as SMILES. We found that this classifier correctly predicts the origin of plant, bacterial or fungal NPs not included in the 60 thousand COCONUT set used for training, as exemplified with the correct prediction of 17 out of 20 newly published NPs. Broader testing of the classifier with further NPs from COCONUT showed limitations for NPs not from plant or microbial origin, such as marine NPs, but it also led to interesting use cases suggesting that the tool might serve as a help to assign NP origin. This concerns in particular NPs isolated from plant but which might in fact be produced by endophytic microorganisms. Such information could be essential when searching for the corresponding biosynthetic genes.

## Methods

### Database preprocessing

The COCONUT database was downloaded. Only the 135,091 (out of 400,837) entries having a taxonomical annotation were selected. The selected subset was further filtered down to the 67,730 entries having an annotation not shorter than ten characters in the DOI field. Then, the taxonomy field was split by commas and match towards the words “plant”/“plants”, “fungi”/“aspergillus”, “bacteria”/“bacillus”/“bacta” to select the NPs with an annotated plant, fungal, or bacterial origin, respectively. The entries common between multiple origins were assigned with the following priority: human > animal > bacteria > fungi > plant > marine. The process led to the selection of 33,772 plant NPs, 15,648 fungi NPs, and 10,751 bacterial NPs with annotated DOI, for a total of 60,171 structures. The number of carbons, oxygen, and nitrogens, the total number of atoms, number of bonds, and TPSA were extracted from the COCONUT annotations. MW, Fsp3, HBD, and HBA count, AlogP, were calculated using RDKit [64]. The presence/absence of a peptide or a glycoside moiety was evaluated using Daylight [65] SMILES arbitrary target specification (SMARTS) language. SMARTS were used with RDKit to identify COCONUT entries containing a dipeptide substructure, defined as “[NX3,NX4+][CH1,CH2][CX3](=[OX1])[NX3,NX4+][CH1,CH2][CX3](=[OX1])[O,N]”, or a containing a glycoside defined as cyclic N- or O-acetal substructure with the SMARTS “[CR][OR][CHR]([OR0,NR0])[CR]”. Substructures were used only for recognizing and labeling peptidic and glycosylated NPs and they were not removed.

### Fingerprint calculation

The 1024 dimensions MinHashed atom pair fingerprint of radius 2 was calculated using the open-source code of MAP4.

### TMAP layout

The indices generated by the MinHash procedure of the MAP4 calculation were used to create a locality-sensitive hashing (LSH) forest [66] of 32 trees. Then, for each structure, the 20 approximate nearest neighbors (NNs) in the MAP4 feature space were extracted from the LSH forest, and the tree layout was calculated. The LSH forest and the minimum spanning tree layout were calculated using the TMAP open-source code. Finally, Fearun [31] was used to display the obtained layout interactively.

### MAP4 SVM implementation

The coconut SUBSET entries used to generate the TMAP were assigned to training or test set with a 50% random split. The SVM was trained using the MAP4 fingerprints of the training set. It utilized a custom kernel that calculates the similarity matrix between two MAP4 fingerprints, where the similarity of fingerprint *a* and fingerprint *b* is calculated (1) counting of elements with the same value and the same index across *a* and *b*, and (2) dividing the obtained value by the number of elements of fingerprint *a*. The class weights were inversely proportional to the class frequency, and the hyperparameter C was optimized using fivefold cross-validation. During the hyperparameter optimization, 20% of the training set was left out as a validation set, and the balanced accuracy of the validation set was maximized. The hyperparameter C was optimized among the values 0.1,1, 10, 100, and 1000, resulting in C = 1. To overcome the intrinsic incapability of SVMs in handling more than two classes, the classifier was implemented using scikit-learn [67] with the “one versus rest” strategy, where in the background one classifier per class is trained and the class is fitted against all the other classes. and all not mentioned hyperparameters were used in their default values. Platt scaling [68], was used to obtain probabilistic prediction values. After the evaluation process, a second version of the MAP4 SVM classifier was trained using both training and test to learn from all curated 60 thousand data points.

### MAP4, ECFP4, RDKit AP, and properties SVMs comparison

The MAP4, ECFP4, and the RDKit AP fingerprints and a set of 11 properties (MW, Fsp3, HBD and HBA count, AlogP, number of carbons, oxygens, and nitrogens, total number of atoms, number of bonds, and TPSA) were used to train four different SVM classifiers in a fivefold cross-validation. For all classifiers, the class weights were inversely proportional to the class frequency, and the hyperparameters were optimized using 10% of the available data (Table 4). For the properties SVM, the 11 values were scaled to zero mean and unit variance.

**Table 4:** Non-default and optimized hyperparameters used in the fivefold cross-validation MAP4, ECFP4, RDKit AP, and properties SVMs comparison

Classifier	Kernel^a^	C^a^	γ^a^
MAP4 SVM	**MAP4**^b^	0.01, 0.1, **1**, 10, 100	–
ECFP4 SVM	**Tanimoto**^c^, Dice^c^	0.01, 0.1, **1**, 10, 100	–
RDKit AP SVM	**Tanimoto**^c^, Dice^c^	0.01, 0.1, 1, **10**, 100	–
Properties SVM	**RBF**^d^	0.01, 0.1, **1**, 10, 100	0.01, 0.1, **1**, 10, 100

^a^Used hyperparameters are reported in bold

^b^Calculates the similarity matrix between two MAP4 fingerprints, where the similarity of fingerprint *a* and fingerprint *b* is calculated (1) counting of elements with the same value and the same index across *a* and *b*, and (2) dividing the obtained value by the number of elements of fingerprint *a*

^c^Ralaivola et al. [69]

^c^Radial basis function (RBF) kernel [70]

### Classifiers evaluation metrics

The F1 score is defined as the harmonic mean of precision and recall:

$$Precision=\frac{TP}{TP+FP}$$

$$Recall=\frac{TP}{TP+FN}$$

$$F1\, score=2\times \frac{(Precision\times Recall)}{(Precision+Recall)}$$

Where TP stands for true positives, TN for true negatives, FP for false positives, and FN for false negatives predicted by the classifier.

The balanced accuracy is defined as:

$$Balanced\, accuracy=\frac{\frac{TP}{TP+FP}+\frac{TN}{TN+FN}}{2}$$

The Matthews correlation coefficient (MCC) is a correlation between the observed and the predicted class and it is defined as:

$$MCC=\frac{TP\times TN-FP\times FN}{\sqrt{(TP+FP)(TP+FN)(TN+FP)(TN+FN)}}$$

### Online MAP4 SVM

The MA4 SVM classifier trained with the whole 60 thousand COCONUT subset is found at https://np-svm-map4.gdb.tools/. The query molecule can be provided as a drawn structure or pasted SMILES in the JSME editor [71]. The given query is canonicalized, chirality information is removed with RDKit, and the MAP4 fingerprint is calculated. To obtain probabilistic prediction values for each class, we use Platt scaling [68].

## Supplementary Information

**Additional file 1.** COCONUT entries for which a publication and an organism name were reported but not the corresponding taxonomical annotation as plant, fungi, or bacteria.

## Data Availability

The code used for the presented work is publicly available at https://github.com/reymond-group/Coconut-TMAP-SVM.

## Abbreviations

AP: Atom pair
COCONUT: Collection of Open Natural Products.
HBA: Hydrogen bond acceptor
HBD: Hydrogen bond donor
LSH: Locality sensitive hashing
MAP4: MinHashed atom pair fingerprint
MCC: Matthews correlation coefficient
ML: Machine learning
MW: Molecular weight
NN: Nearest neighbor
NP: Natural product
SMARTS: SMILES arbitrary target specification
SMILES: Simplified molecular-input line-entry system
SVM: Supported vector machine
TMAP: Tree-map
TPSA: Topological polar surface area
